# Aducanumab in Alzheimer’s Disease: A Comparative Study of Its Effects on Dementia and Mild Cognitive Impairment

**DOI:** 10.7759/cureus.75907

**Published:** 2024-12-17

**Authors:** Andrea Medel Sánchez, Arturo Ortiz Hernández, Ramiro A Moreno Moreno, Diana Salas López, Luz E Madrigal Gómez, Anna K Dominguez Ibarra, Beatriz A Gutiérrez Rojas, Cesar O Garcia Navarro, Gerardo T Moreno Becerril, Mauricio Montelongo Quevedo, Jose R Flores Valdés

**Affiliations:** 1 General Practice, Universidad de Guanajuato, Leon, MEX; 2 General Medicine, Universidad Autónoma de Tamaulipas, Ciudad Victoria, MEX; 3 General Medicine, Universidad Autónoma de Nuevo León, San Nicolás de los Garza, MEX; 4 General Practice, Universidad Hipócrates, Acapulco, MEX; 5 General Practice, Universidad Autónoma de Guadalajara, Guadalajara, MEX; 6 General Practice, Universidad Autónoma de Ciudad Juárez, Ciudad Juárez, MEX; 7 General Practice, Universidad Juárez del Estado de Durango, Gomez Palacio, MEX; 8 General Practice, Universidad de Guadalajara, Guadalajara, MEX; 9 General Practice, Universidad Autónoma del Estado de México, Toluca, MEX; 10 General Medicine, Universidad Autónoma de Guadalajara, Guadalajara, MEX; 11 Research, Oncology Consultants PA, Houston, USA

**Keywords:** aducanumab, alzheimer's disease, amyloid-related imaging abnormalities, amyloid-β, biomarkers, dementia, monoclonal antibody, systematic review

## Abstract

Alzheimer's disease (AD) is the leading cause of dementia, characterized by progressive cognitive decline. Cholinesterase inhibitors are commonly used to manage symptoms but have limited efficacy as the disease progresses. Aducanumab, a monoclonal antibody targeting amyloid-β (Aβ) plaques, has emerged as a novel therapeutic approach. Despite its Food and Drug Administration (FDA) approval, its efficacy and safety remain contentious, particularly following the European Medicines Agency's (EMA's) rejection. This systematic review aims to evaluate the efficacy, safety, and clinical outcomes of aducanumab in treating mild AD. Adhering to Preferred Reporting Items for Systematic Reviews (PRISMA) 2020 guidelines, we conducted a comprehensive search of PubMed and Science Direct databases, including randomized controlled trials (RCTs), cohort studies, and case-control studies focusing on aducanumab versus placebo in mild AD. Studies were screened based on predefined inclusion and exclusion criteria, and data were extracted on clinical outcomes, biomarkers, and neuroimaging markers. The risk of bias was assessed using the Cochrane Handbook and Newcastle-Ottawa Scale. Out of 967 identified records, seven studies met the inclusion criteria. Findings indicated a dose-dependent reduction in Aβ plaques with aducanumab, but clinical outcomes varied. High-dose aducanumab (10 mg/kg) demonstrated significant improvements in some studies but not others. Adverse events, notably amyloid-related imaging abnormalities (ARIA), were frequent, especially at higher doses. The studies exhibited heterogeneous treatment effects and underscored the potential of cerebrospinal fluid biomarkers as an alternative to amyloid positron emission tomography (PET) scans. Aducanumab shows promise in reducing Aβ plaques and has potential clinical benefits at high doses; however, its safety profile, particularly concerning ARIA, remains a significant concern. The variability in clinical efficacy highlights the need for further research to optimize dosing regimens and identify patient populations most likely to benefit from treatment. Future studies should focus on refining treatment protocols and exploring alternative biomarkers to improve therapeutic outcomes for AD.

## Introduction and background

Alzheimer's disease (AD) is a progressive, irreversible brain disorder and the most common form of dementia [1]. Approximately 55 million people worldwide live with dementia, and Alzheimer's accounts for 60% to 80% of these cases [1,2]. Although the exact cause of this disorder is still unknown, it is observed that among the most frequent anatomical features is the accumulation of amyloid plaques and tau proteins in various brain regions, leading to neuronal damage and their connections, resulting in cognitive impairment that affects the patient's thinking, memory, and behavior [2,3].

It is noteworthy that according to the WHO, by 2050, the number of people with dementia could increase to 152 million, influenced by the increasing life expectancy of the population, which will entail significant costs in medical, social, and caregiver support services, diminishing quality of life for both patients and their families [4,5].

Symptoms of mild to moderate AD are treated with cholinesterase inhibitors such as galantamine, rivastigmine, and donepezil, aimed at preventing the breakdown of acetylcholine and reducing cognitive and behavioral symptoms. Unfortunately, as we age, the brain produces less acetylcholine, reducing the effectiveness of these medications [6]. Aducanumab is a new treatment aimed at addressing the early stages of Alzheimer's in the United States. It was approved by the Food and Drug Administration (FDA) through the accelerated approval pathway in 2021 due to the importance and severity of the disease. It is an intravenously administered recombinant monoclonal antibody developed from the derivation of a library of blood lymphocytes collected from cognitively intact or slowly cognitively declining healthy elderly individuals. In contrast to previous drugs, it is specifically directed toward the N-terminus of the beta-amyloid peptide rather than just the symptoms of the disease [3,7,8].

While the future of aducanumab appears promising in the United States, the European Medicines Agency (EMA) decided to withdraw the marketing authorization application for aducanumab, citing unclear effects and clinical improvement related to the drug, as anomalies such as swelling and bleeding had been revealed in images [9]. Given the controversial approval of the drug aducanumab by the United States FDA, followed by its withdrawal from the market by the EMA, there is a need to conduct a systematic review of the available evidence. Justified to reduce the gaps in information present in the current scientific literature and to provide clarity and coherence in the evaluation of the efficacy and safety of aducanumab.

For the past 30 years, the amyloid hypothesis has been proposed, suggesting that the accumulation of amyloid-β protein is the cause of AD; however, this hypothesis was considered unlikely due to many years of study without significant changes. The introduction of aducanumab into Alzheimer's treatment research is a promising step, but it is imperative to note that the use of this medication as therapy could also be harmful [10]. Therefore, it is important to monitor and recognize if the drug is beneficial, evaluating changes at the brain level, as amyloid-related imaging abnormalities (ARIA) such as cerebral edema, sulcal effusion, or hemosiderin deposits secondary to hemorrhage have been observed in magnetic resonance imaging [11].

This systematic review aims to compile and synthesize relevant evidence on the efficacy, safety, and effects found in the use of an anti-amyloid monoclonal antibody (aducanumab) in the treatment of Alzheimer's in mild stages.

## Review

Methods

This study adhered to the Preferred Reporting Items for Systematic Reviews (PRISMA) 2020 guidelines and evidence-based medicine to ensure a comprehensive and systematic approach to our review [12,13].

Searching methods

Stringent inclusion and exclusion criteria were established to ensure the inclusion of only high-quality studies. The exclusion criteria were rigorously applied to maintain the quality and relevance of the studies analyzed. Studies that did not focus on comparing aducanumab with placebo in the treatment and better clinical outcomes in patients with Alzheimer’s were excluded. Additionally, studies that were not available in full text or could not be obtained via interlibrary loans were also excluded.

The literature search was conducted across multiple databases: PubMed (Table 1) and Science Direct (Table 2). The search strategy employed Medical Subject Headings (MESH) terms and free-text terms relevant to our research question. The article selection process was guided by a PRISMA flowchart. This meticulous approach enabled the creation of a homogeneous dataset, facilitating a more accurate and reliable analysis of the results.

**Table 1:** PubMed

Search strategy	Results
((("alzheime s"[All Fields] OR "alzheimer disease"[MeSH Terms] OR ("alzheimer"[All Fields] AND "disease"[All Fields]) OR "alzheimer disease"[All Fields] OR "alzheimer"[All Fields] OR "alzheimers"[All Fields] OR "alzheimer s"[All Fields] OR "alzheimers s"[All Fields]) AND "alzheimer disease"[MeSH Terms]) OR ("alzheimer disease"[MeSH Terms] OR ("alzheimer"[All Fields] AND "disease"[All Fields]) OR "alzheimer disease"[All Fields] OR ("alzheimers"[All Fields] AND "disease"[All Fields]) OR "alzheimers disease"[All Fields]) OR ("alzheimer disease"[MeSH Terms] OR ("alzheimer"[All Fields] AND "disease"[All Fields]) OR "alzheimer disease"[All Fields] OR ("familial"[All Fields] AND "alzheimer"[All Fields] AND "disease"[All Fields]) OR "familial alzheimer disease"[All Fields]) OR ("alzheimer disease"[MeSH Terms] OR ("alzheimer"[All Fields] AND "disease"[All Fields]) OR "alzheimer disease"[All Fields] OR ("acute"[All Fields] AND "confusional"[All Fields] AND "senile"[All Fields] AND "dementia"[All Fields]) OR "acute confusional senile dementia"[All Fields]) OR (("primaries"[All Fields] OR "primary"[All Fields]) AND ("senile"[All Fields] OR "seniles"[All Fields] OR "senility"[All Fields]) AND ("degenerative"[All Fields] OR "degeneratively"[All Fields] OR "degeneratives"[All Fields]))) AND ("aducanumab"[Supplementary Concept] OR "aducanumab"[All Fields] OR "aducanuma*"[All Fields]) AND ("placebo effect"[MeSH Terms] OR ("placeboes"[All Fields] OR "placebos"[MeSH Terms] OR "placebos"[All Fields] OR "placebo"[All Fields]) OR "placebo"[Title/Abstract]) AND ((("randomized controlled trial"[Publication Type] OR "controlled clinical trial"[Publication Type] OR "clinical trials as topic"[MeSH Terms:noexp] OR "trial"[Title] OR "random*"[Title/Abstract] OR "placebo*"[Title/Abstract]) AND (("clinical"[Title/Abstract] AND "trial"[Title/Abstract]) OR "clinical trials as topic"[MeSH Terms] OR "clinical trial"[Publication Type] OR "random*"[Title/Abstract] OR "random allocation"[MeSH Terms] OR "therapeutic use"[MeSH Subheading])) OR ("cohort studies"[MeSH Terms] OR "case-control studies"[MeSH Terms] OR "comparative study"[Publication Type] OR "risk factors"[MeSH Terms] OR "cohort"[Text Word] OR "compared"[Text Word] OR "groups"[Text Word] OR "case control"[Text Word] OR "multivariate"[Text Word]))	32

**Table 2:** Science Direct

Search strategy	Results
Alzheimer Disease OR Alzheimer dementia AND mild cognitive impairment AND Aducanumab AND placebo AND randomized controlled trials NOT meta-analysis NOT systematic reviews	967

Selection criteria

Types of Participants

Selection criteria were established for this study for specific participants, which included individuals presenting with mild cognitive impairment (MCI) attributable to AD or mild AD dementia, individuals between the ages of 50 and 85 years, irrespective of gender, individuals who satisfy the clinical criteria for AD: either a Clinical Dementia Rating-Global Score of 0.5, verifiable evidence of cognitive impairment during screening, or a Mini-Mental State Examination score ranging from 20 to 24, it is required to possess a positive amyloid positron emission tomography (PET) scan, in case of utilizing medications to address symptoms associated with AD, the dosages must have remained stable for a minimum of eight weeks before the first screening visit.

On the other hand, among the exclusion criteria, we find patients with any additional medical or neurological conditions that may potentially contribute to cognitive decline, such as stroke, transient ischemic attack (TIA), or unexplained loss of consciousness within the past year, clinically significant psychiatric illness in the past six months, signs of compromised kidney or liver function, have experienced a significant systemic illness or infection within the past 30 days, significant brain hemorrhage, bleeding disorder, cerebrovascular abnormalities, or alcohol or substance abuse within the last year, past episodes of unstable angina, heart attack, severe chronic heart failure, or any notable conduction abnormalities within the year before the screening, have been diagnosed with human immunodeficiency virus (HIV), any reasons that would prevent undergoing brain magnetic resonance imaging (MRI) or PET scans, alcohol or drug misuse within the last year.

Types of Intervention

This study focuses on patients who received FDA-approved anti-Aβ monoclonal antibodies (aducanumab) and patients who were administered intravenously with aducanumab at doses ranging from 1 mg/kg to 10 mg/kg. On the other hand, the following group is outside the scope of our primary investigation: patients with a history of treatment involving any medication other than aducanumab for MCI due to AD or mild dementia associated with AD; patients treated with anti-Aβ lecanemab, if taking Alzheimer's medication, had to have been on a stable dose for at least eight weeks before the screening and be taking antiplatelets (except prophylactic doses or less of aspirin).

Types of Studies

For this study, only double-blind placebo-controlled randomized controlled trials (RCTs), cohort studies, and case-control studies were considered, excluding in turn case reports, expert opinions, case series, letters to the editor, reviews, and meta-analyses, as well as articles written in a language other than English or Spanish. Studies with incomplete data were excluded. Studies without full text available were not considered.

Type of Outcomes

Inclusion criteria used for outcomes encompassed clinical, biomarker, and neuroimaging markers. For clinical outcomes, the Clinical Dementia Rating Sum of Boxes (CDR-SB) was selected, in addition to the Alzheimer's Disease Cooperative Study-Activities of Daily Living Scale for Mild Cognitive Impairment (ADCS-ADL-MCI), the Alzheimer's Disease Composite Score (ADCOMS), or the Alzheimer's Disease Assessment Scale-Cognitive portion (ADAS-Cog). Our neuroimaging criterion was the ratio of the standardized amyloid uptake value by PET (PET SUVr) and, finally, cerebrospinal fluid (CSF) levels of Aβ1-42, phosphorylated tau181 (p-tau), and total tau (t-tau); the plasma Aβ42/40 ratio or plasma tau181 served as biomarkers. On the other hand, the exclusion criteria for this review were studies that did not meet at least one inclusion outcome.

Selection of studies, data extraction, and screening

Two reviewers (AKDI, COGN) employed Rayyan to screen titles and abstracts. Subsequently, a third independent reviewer (RAMM) verified the relevance of the studies according to predefined inclusion and exclusion criteria. Following this, a detailed full-text analysis was performed, where two other reviewers (RAMM, COGN) independently selected trials based on the same inclusion and exclusion criteria. Disagreements in this stage were similarly resolved through consensus and with the assistance of the third review author (BAGR).

The studies retrieved during the searches will be screened for relevance, and those identified as being potentially eligible will be fully assessed against the inclusion/exclusion criteria and selected or rejected as appropriate.

Data will then be extracted from the studies selected for inclusion, as follows: study name, publication year, study period, study design, mean age, female percent, biopsy route, surgical procedure for tumor excision and role of minimally invasive techniques, conservative management among myxomas, early and late mortality, and long-term recurrence data.

Data evaluation: assessment of risk of bias

Our evaluation followed the criteria outlined in the Cochrane Handbook. The Newcastle-Ottawa Scale (NOS) was used for case-control studies and cohort studies. Two independent reviewers assessed the risk of bias in each study, considering the specific criteria and guidelines of the respective tools. Discrepancies between reviewers were resolved through discussion with a third, blinded reviewer (JRFV). According to the Cochrane Handbook for Systematic Reviews of Interventions and NOS guidelines, the methodological aspects of the cohort and case-control studies were categorized as having a low, high, or unclear risk of bias. Details regarding any changes in the quality of evidence, either downgrading or upgrading, were transparently presented in the summary of findings table, along with explanations for each bias assessment.

Results

We conducted a systematic literature review to evaluate the effects of aducanumab on dementia and MCI in AD. Our search included studies published between 2016 and 2024 and focused on articles indexed in the PubMed database. We used a comprehensive set of keywords, including 'Alzheimer’s disease,' 'dementia,' 'aducanumab,' 'placebo,' and 'mild cognitive impairment,' to identify relevant research.

**Figure 1:**
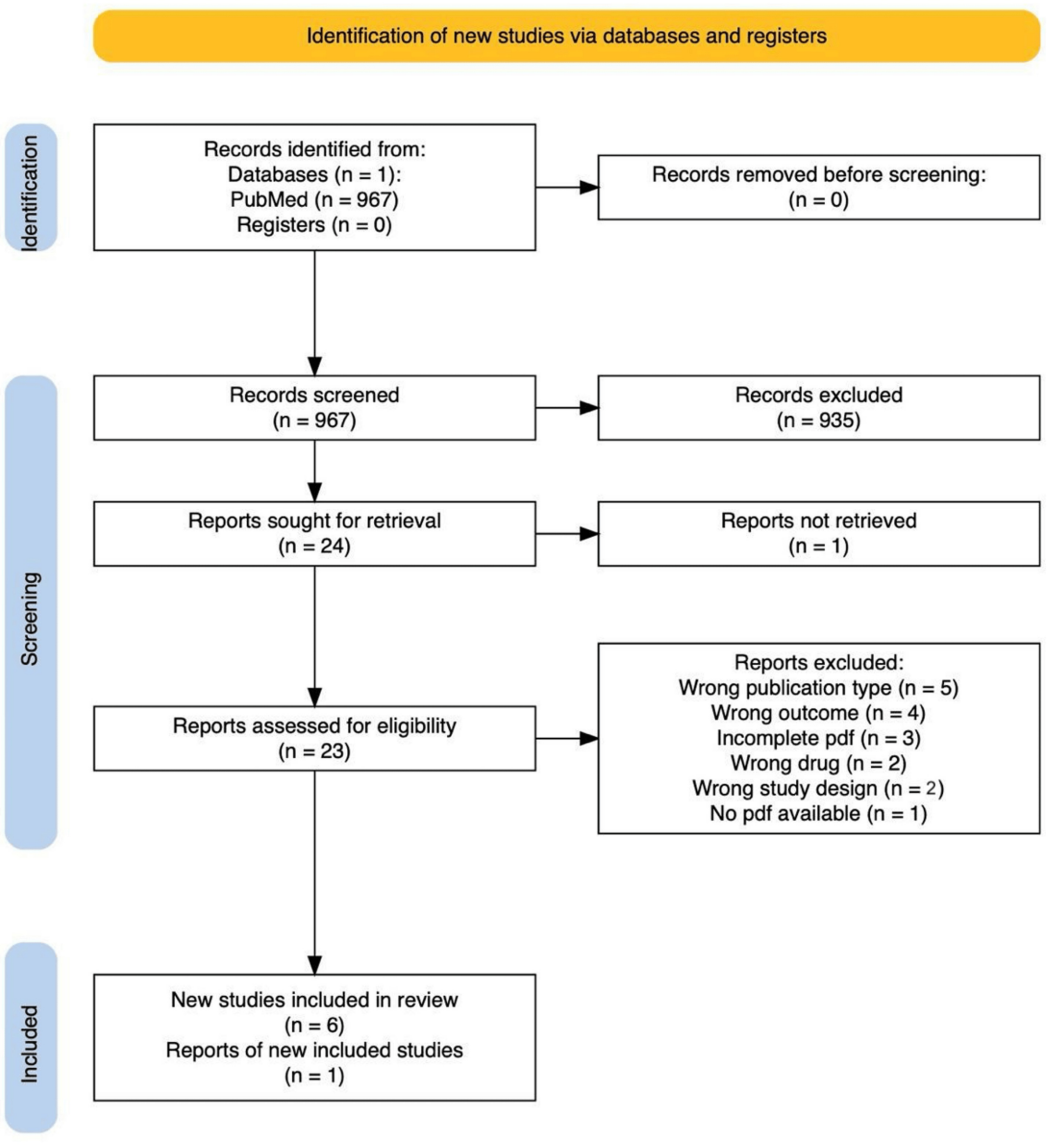
PRISMA flow chart The PRISMA [12] flow diagram outlines the process of study selection for a systematic review. Initially, 967 records were identified from PubMed. After screening, 935 records were excluded. Of the 24 reports sought for retrieval, one was not retrieved, leaving 23 reports assessed for eligibility. Subsequently, 17 reports were excluded due to wrong publication type (n=5), wrong outcome (n=4), incomplete PDFs (n=3), wrong drug (n=2), wrong study design (n=2), and no PDF available (n=1). Finally, 6 studies were included in the review. PRISMA: Preferred Reporting Items for Systematic Reviews and Meta-Analyses

As depicted in our PRISMA flow diagram [12], our initial search yielded 967 records (Figure 1). After removing duplicates and screening titles and abstracts, we thoroughly evaluated 24 full-text articles for eligibility. Ultimately, seven studies met our inclusion criteria and were included in this comprehensive review. Additionally, we incorporated a randomized controlled trial (RCT) sourced from Springer Nature, which aligned with our inclusion criteria and followed the previously mentioned search strategy.

**Figure 2:**
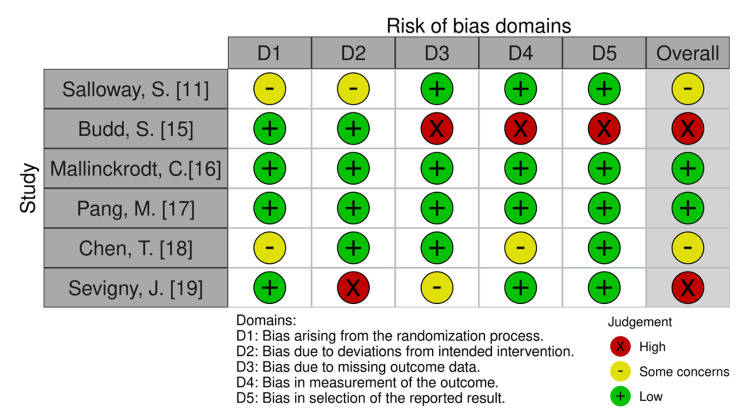
Risk of bias The table displays the risk of bias [14] assessment for seven studies across five domains: bias arising from the randomization process (D1), bias due to deviations from intended intervention (D2), bias due to missing outcome data (D3), bias in the measurement of the outcome (D4), and bias in the selection of the reported result (D5). Judgment categories are represented by colors, with green (+) indicating a low risk of bias, yellow (-) indicating some concerns, and red (X) indicating a high risk of bias. The overall risk of bias is also summarized for each study. The studies by Mallinckrodt and Pang show a low overall risk, Salloway and Chen have some concerns, while Sevigny and Budd have a high overall risk of bias.

Our assessment of the risk of bias, summarized in Figure 2, indicated a low risk of bias in two studies, a moderate risk in two studies, and a high risk in two studies, primarily due to deviations from the intervention, missing outcome data in some cases, as well as inadequate measurement and selection of outcomes that did not meet the required quality standards.

The systematic review focuses on the effectiveness and safety of aducanumab in treating AD, as summarized in Table 3. The studies included in the review span multiple countries, providing a broad spectrum of clinical scenarios associated with aducanumab treatment. Budd et al. (2022) [15] conducted a multicentric randomized controlled trial (RCT) with a total sample size of 3,285 patients, divided into 2,192 receiving aducanumab and 1,093 receiving a placebo. Patients aged 50-85 received varying doses of aducanumab (low dose: 3 or 6 mg/kg; high dose: 10 mg/kg) over 18 months. The study noted that the global EMERGE trial demonstrated clinical benefits at high doses, while the ENGAGE trial did not. Biomarker analyses revealed modifications in disease pathology, although clinical outcomes varied. The primary adverse event was amyloid-related imaging abnormalities with edema (ARIA-E). Salloway et al. (2021) [11] also reported on the EMERGE and ENGAGE trials, highlighting that 41.3% of participants treated with 10 mg/kg aducanumab experienced ARIA, with over a third developing edema and about a quarter showing symptoms. Additionally, microhemorrhages and superficial siderosis were noted as adverse events.

**Table 3:** General outcomes ApoE: apolipoprotein E; ARIA-E: amyloid-related imaging abnormalities with edema; ARIA: amyloid-related imaging abnormalities; FDA: Food and Drug Administration; ITR: individual-level treatment response; CDR-SB: Clinical Dementia Rating Scale Sum of Boxes; AD: Alzheimer disease; ADAS-CS: Alzheimer's Disease Assessment Scale - Cognitive Subscale; MMSE: Mini-Mental State Examination; ADCS-ADL-MCI: Alzheimer's Disease Cooperative Study - Activities of Daily Living Scale for use in Mild Cognitive Impairment; NPI: intelligence-free numeric identifier; LTE: long-term evolution; PC: placebo-controlled; CDR-SB: Clinical Dementia Rating Scale Sum of Boxes

Author	Year	Country	Study design	Total sample size	Total sample intervention (aducanumab)	Total sample size (placebo)	Total age mean/SD at diagnosis	Aducanumab dose	# Amyloid-related imaging abnormalities (ARIA) in aducanumab	# Amyloid-related imaging abnormalities (ARIA) in placebo	Follow up	Key points
Budd et al. [15]	2022	Multicentric	RCT	3285	2192	1093	50-85 years	Receive aducanumab low dose (3 or 6 mg/kg target dose), high dose (10 mg/kg target dose)	2192	1093	18 months	In the global EMERGE and ENGAGE trials, 3285 mild Alzheimer's patients received aducanumab or a placebo for 76 weeks. Allocation factored in treatment site and ApoE ε4 status. The trials ended early to prevent ineffective treatments, with no validity compromise. EMERGE's high dose showed clinical benefits, unlike ENGAGE. Biomarker analyses revealed disease pathology modification, though clinical outcomes differed. ARIA-E was the primary adverse event. Dosing variability likely impacted clinical outcome disparity
Salloway et al. [11]	2021	Multicentric	RCT	3285	2198	1087	70.4 (7.45) years	3, 6, 10 mg/kg	2198	1087	15 months	In the EMERGE and ENGAGE trials, among 3285 participants, 41.3% of those treated with 10 mg/kg aducanumab experienced ARIA, with over a third developing edema and about a quarter of those showing symptoms. Additionally, 19.1% had microhemorrhages and 14.7% had superficial siderosis.
Mallinckrodt et al. [16]	2023	Multicentric	RCT	3312	2208	1104	67.5 years	It was divided into 3, 6, and 10 mg/kg	672 ENGAGED and 655 in EMERGE	164 ENGAGNE and 163 EMERGE	19 months	The discordance between EMERGE and ENGAGE in patients receiving high doses of aducanumab is secondary to the lack of methods to quantify rapid disease progressors and the administration of inappropriate doses.
Pang et al. [17]	2023	United States of America	RCT	587	299	288	50 to 85 years	Low dose (3 or 6 mg/kg target dose), high dose (10 mg/kg target dose), or placebo IV infusion.	299	288	4 weeks over 76 weeks	Using an individual-level treatment response (ITR) score model, researchers identified variability in high-dose aducanumab's efficacy compared to placebo in reducing CDR-SB decline in the EMERGE trial. Those benefiting most had specific baseline traits: lower hippocampal and medial temporal cortical volumes, older age, higher plasma p-tau181 levels, shorter time since AD symptom onset and diagnosis, and a higher microhemorrhage prevalence. This cohort exhibited enhanced aducanumab treatment effects across cognitive (MMSE, ADAS-Cog13), functional (ADCS-ADL-MCI), and neuropsychiatric (NPI-10) domains, not used in model training. However, lacking independent validation, these findings remain hypothesis-generating.
Chen et al. [18]	2024	United States of America	RCT	196	148	48	NR	Intravenous aducanumab (1, 3, 6, or 10 mg/kg)	148	48	48 months	This report combines data from PRIME's placebo-controlled (PC) and long-term extension (LTE) phases. ARIA-E, mainly occurring in the initial 24 weeks of aducanumab treatment, was transient and asymptomatic in PC phase participants. Similar trends were noted in those switching from placebo to aducanumab in the LTE phase. Dose titration mitigated ARIA-E risk in APOE ε4 carriers. At 10 mg/kg with titration, stability was reached by month 24 over 48 months. Amyloid plaque levels stabilized, notably after 36 months, with a reduction seen in LTE participants switching from placebo. The findings suggest aducanumab's potential impact on amyloid reduction, albeit warranting further validation.
Sevigny et al. [19]	2016	United States of America	RCT	165	125	40	72.6 years	It was divided into 1, 3, 6, and 10 mg/kg	42	1	13 months	The administration of aducanumab increases the elimination of brain Aβ plaques. The outcome demonstrates that its effects are dose-dependent and were demonstrated through scales such as the CDR-SB and MMSE.

Mallinckrodt et al. (2023) [16] provided further insights from the EMERGE and ENGAGE trials involving 3,312 patients. The study emphasized the discordance between the trials, attributing it to insufficient methods to quantify rapid disease progressors and inappropriate dosing. The study reported ARIA as a significant adverse event, particularly at higher doses. Pang et al. (2023) [17] explored individual-level treatment responses (ITRs) in a cohort of 587 patients, highlighting variability in high-dose aducanumab efficacy. The study identified specific baseline traits associated with better treatment outcomes, although these findings are preliminary and require further validation. Chen et al. (2024) [18] reported on a study combining data from the PRIME trial's placebo-controlled and long-term extension phases, involving 196 patients. The study observed transient and asymptomatic ARIA-E mainly in the initial 24 weeks of treatment. Dose titration appeared to mitigate ARIA-E risk in APOE ε4 carriers, with stability reached by month 24. Sevigny et al. (2016) [19] conducted an RCT with 165 patients, demonstrating that aducanumab dose-dependently reduced brain amyloid plaques. The study showed positive outcomes on cognitive scales such as CDR-SB and MMSE.

This review consolidates data from various global sources, providing insights into aducanumab's efficacy and safety profile in AD treatment. Future studies could focus on standardizing treatment protocols to improve outcomes and reduce complications globally. The results section encapsulates detailed findings and study characteristics, offering a comprehensive snapshot of the international landscape of aducanumab research.

Budd et al. (2022) [15] reported significant differences in outcomes between the EMERGE and ENGAGE trials. In the EMERGE trial, high-dose aducanumab significantly reduced AD symptoms compared to placebo (difference of -0.39 (95% CI, -0.69 to -0.09; p=0.012; 22% reduction)). Conversely, the ENGAGE trial showed no significant difference (difference of 0.03 (95% CI, -0.26 to 0.33; p=0.833; 2% increase)). Both trials observed a dose- and time-dependent reduction in Alzheimer's pathophysiological markers. Salloway et al. (2021) [11] noted a higher incidence of ARIA-E in the 10 mg/kg aducanumab group (35.2%) compared to the 6 mg/kg group (21.2%), the 3 mg/kg group (29.5%), and the placebo group (2.7%). Additionally, ARIA-E was more common in ApoE ε4 carriers (43.0%) than non-carriers (20.3%) within the 10 mg/kg group. Chen et al. (2024) [18] observed that in the PRIME study, APOE ε4 carriers had a lower incidence of ARIA-E when titrated to 10 mg/kg aducanumab (35%) compared to fixed doses of 6 mg/kg (43%) or 10 mg/kg (55%). The incidence of ARIA-H microhemorrhages during the titration period was 22% in the fixed-dose group at 10 mg/kg, lower in the titration group at 10 mg/kg (17%). Mallinckrodt et al. (2023) [16] conducted a post hoc analysis of four areas, revealing that high-dose aducanumab outcomes in the ENGAGE trial were influenced by a small subset of patients with rapid disease progression and lower exposure to the 10 mg/kg target dose. Patients who received the full target dose showed more significant treatment effects, indicating this dosing regimen's potential effectiveness.

Sevigny et al. (2016) [19] reported results from the PRIME phase 1b randomized trial with 165 Alzheimer's patients. Monthly infusions of aducanumab at doses of 1, 3, 6, or 10 mg/kg resulted in significant reductions in PET standard uptake value ratio (SUVR) composite scores at 54 weeks (p<0.001). The 10 mg/kg dose group approached the quantitative cut-point, discriminating between positive and negative scans. Clinical assessments, including CDR-SB and MMSE, showed dose-dependent slowing of disease progression with the greatest effects at 10 mg/kg (p<0.05 vs. placebo). Delrieu et al. (2019) [20] discussed the impact of aducanumab (ADUHELMTM) on amyloid plaque removal in phase Ib and III trials. Higher exposure to aducanumab correlated with greater effects on cognitive decline and amyloid plaque load, potentially influencing future therapeutic trial designs. Despite FDA approval in the United States, there is uncertainty about its global applicability. Pang et al. (2023) [17] identified heterogeneous treatment effects (HTE) in the CDR-SB. The top 25% of patients with the highest ITR scores had a CDR-SB benefit of 0.79 points greater than the remaining 75% (p=0.0020). Key predictors of response included lower hippocampal volume and higher plasma phosphorylated tau 181 levels. Chen et al. (2024) [18] also reported on the PRIME study, highlighting that APOE ε4 carriers experienced a higher incidence of ARIA-E in the placebo-controlled period across all aducanumab dose groups. The incidence of ARIA-E was lower in the titrated 10 mg/kg group (35%) compared to fixed doses of 6 mg/kg (43%) and 10 mg/kg (55%). Additionally, amyloid plaque levels declined significantly, particularly in the 10 mg/kg fixed-dose group, demonstrating substantial amyloid reduction.

This review consolidates findings from various global studies, emphasizing the variability in aducanumab's efficacy and safety across different doses, patient populations, and biomarkers. Future research should aim to standardize treatment protocols to optimize outcomes and reduce adverse effects. The results section provides detailed study characteristics and results, offering a comprehensive overview of aducanumab research.

Discussion

AD presents a formidable challenge to global public health efforts, as finding a cure remains elusive. Monoclonal antibody drugs directed at Aβ proteins like aducanumab have emerged intending to slow AD progression. Despite the FDA approval as treatment, the lack of knowledge about the proper effect on the use in the early stages of the illness continues to be subject to ongoing scrutiny.

This systematic review provides valuable insights into this debate, underlining the importance of assessing bioavailability at low and high drug doses. Our study aims to evaluate the effectiveness and safety of aducanumab in patients with AD experiencing mild dementia, comparing its treatment benefits to a placebo. Our qualitative analysis indicates that patients treated with aducanumab at low doses (ranging from 1 mg/kg to 3 mg/kg) exhibit reduced accumulation of Aβ-amyloid plaque in the brain and reduced adverse effects after administration. In contrast, it exhibits notable side effects, particularly the high incidence of ARIA at high doses. This has raised doubts about its overall benefit.

Our findings point to previous studies, such as the 2024 meta-analysis by Wu et al. [21], and a phase III clinical trial by Mallinckrodt et al. [16], which was used to test the efficacy of aducanumab. These included two large trials, ENGAGE with 1638 participants and EMERGE with 1647 participants. The results of these two articles were concordant with each other for the most part. However, the high-dose results were discordant. While high-dose aducanumab showed significant treatment effects on primary and secondary endpoints in EMERGE, in ENGAGE, such effects were not observed [15]. Mean differences from placebo in biomarkers and clinical outcomes were similar for low doses and declined for high doses in EMERGE. However, a recent meta-analysis reviewed two independent studies representing 4471 patients. The studies consisted of RCTs with 2190 patients in treatment with aducanumab and 2281 patients in the placebo group. Patients who received treatment had statistical improvement in clinical and neuroimaging outcomes, including CDR-SB (p=0.01), increased Aβ1-42 in CSF (p=0.002), and decreased P-Tau in CSF (p<0.00001) [21].

Moreover, the data provided by the PRIME study show information on the long-term efficacy and safety of treatment with aducanumab. The dose- and time-dependent decrease in amyloid plaque levels and improvements in cognitive measures such as CDR-SB and MMSE support the notion that aducanumab effectively targets and removes brain Aβ plaques. However, the observed plateau in amyloid plaque levels over time raises questions about the durability of treatment effects and the need for continued follow-up [19].

The use of ITR models by Pang et al. 2024 [17] revealed HTE, suggesting that certain baseline characteristics may predict greater benefit from aducanumab. This proposes further investigation of precision medicine approaches to personalize treatment decisions for patients with early AD [17].

We acknowledge several limitations in our study. First, the number of cohort studies we included was limited, and their sample sizes varied considerably. Additionally, our analysis focused on the experimental group at a single dose (1-10 mg), neglecting to consider the potential impact of different dosages on outcomes. In consequence, conducting direct comparisons was a challenge. The predominance of randomized control studies and the lack of high-quality cohort studies could have induced bias.

The FDA has approved the first monoclonal antibody therapy that targets the fundamental pathophysiology of the disease. Since then, different studies have been looking for the best strategy for a better approach to reducing the development of side effects after administration for a long time.

Improvements in pharmacological agents and advanced imaging techniques hold promise for further enhancing the efficacy of aducanumab in the treatment of AD. The studies evaluated had various risks of bias, underscoring the need for additional high-quality research to strengthen the evidence for safety in AD.

## Conclusions

In the field of AD treatment, conducting a systematic review presents unique challenges and limitations due to the wide range of clinical presentations, study methodologies, interventions, and methods of outcome assessment. Despite significant investments in research, the efficacy of AD treatments remains limited. Although aducanumab has shown promise in reducing the accumulation of β-amyloid plaque in the brain, questions about its clinical efficacy persist. In addition, notable adverse effects, particularly the high incidence of ARIA, have raised doubts about its overall benefit. Other research underscores the need to better understand individual responses to anti-amyloid therapy and suggests that certain baseline characteristics may predict a more favorable outcome with aducanumab. All the above led its manufacturer to discontinue production of aducanumab two years after the controversial FDA approval. Nevertheless, the search for new therapeutic avenues continues, driven by the aspiration to discover more effective interventions against AD.

In summary, although aducanumab has demonstrated its potential as a treatment for AD, its use is accompanied by important safety considerations and variability in response to treatment. Despite the difficulties, advances in research offer hope for addressing the global burden of AD. Future research efforts should prioritize refining patient selection criteria, optimizing dosing regimens, and exploring alternative biomarkers to facilitate treatment approaches.
